# Supplementary material for: Chromothripsis during telomere crisis is independent of NHEJ, and consistent with a replicative origin
Source: Genome Res. 2019 May;29(5):737–49. doi: 10.1101/gr.240705.118 (PMC6499312; doi:10.1101/gr.240705.118)
Supplement: Supplemental Material [file supp_gr.240705.118_Supplemental_file_1.zip › contigs/annotated_contigs/DB73/contig.2.DB73_length_766_mean_cov_7.82637075718.docx]

**DB73_length_766_mean_cov_7.82637075718**

```
TGATTAACAAAGTGTTTTCACATCTATTGTGGCCTCTCATAAATCCTGGACAAAAATTAGGGTAAGCATGTTATTCCTGTTTGTAGAGA
 >chr1:50286357-50286672 - E=1e-177
AGGAAGTTGAAGAAATTAAGATTTAGAGAAGTTGTGACTTGACTAAGTGCGCTTAACTAATAAGTGGCAGAATCTGGAGTTCAGCCCAG

GCCTACCGGACCTCAAAACCTGTTCTTATACATCTTAGTTAACTTTTGGTTATTCAGCATTTATATGTTTATTTCCTGGCTCATTATTA

GCAACTAGAGCATTTCCAAGTTGAAGTACAAGCTAATAACCAG|TCTGA|ATCCTGAAAGCATACTCTAATTATGATGGTATTATTCTG
                                            >chr1:50281492-50281948 - E=1e-262
GTTGTATCTGTAAAGTAGTACTAGTGAGAGTTAAGGGTGGTAATGATAAGAGCTGAGCATGCTGCCTCAGGTAATCTCAATGACTCAGG

AGCCTGAGGCAGAAGGACCACTTGAGTCCAGGACTTTGAGACCAGCCTGGGCAACATAGCAAGACCCCATCTCTTAAAAAAATACATAG

CCAGGCATGGTGGCATATGCCTGTAAACCCCAGTGACTGGGGAGGCTGATGGGGGAGGATTGCTTCAGCCTAGGAGCTTGAGGCTGCAG

TGAGTCATGATCCTGTCATTGCAATCCATCCTGGGCAACAGAGTAAGACCTCCCACTGAAAAAAAAGATGGTAATGATTGAAATTAGGT

GAGTGTTTCCCTACCTAAGAAACAGCTAGTTTTCAAAAGACTTGAATAGACATTTC
```
